# Establishment of a Lassa Fever Specimen Biobank in Nigeria

**DOI:** 10.4269/ajtmh.24-0527

**Published:** 2025-05-27

**Authors:** Hanesh F. Chi, Johnson Etafo, Fritz Fonkeng, Gbenga-Ayeni Bosede Olufunke, Ronke Ireneh, Chukwuyem Abejegah, Sampson Owhin, Stella Somiari, Aurélia Vessière, Daniel G. Bausch, Imane El Idrissi, Warren Fransman, Cassandra Kelly-Cirino, Emmanuel Agogo, Devy M. Emperador, Nelson A. Adedosu

**Affiliations:** ^1^ FIND, Geneva, Switzerland;; ^2^ Federal Medical Centre Owo, Owo, Nigeria;; ^3^ ITSI Biosciences, Johnstown, Pennsylvania;; ^4^ London School of Hygiene and Tropical Medicine, London, United Kingdom

## Abstract

Despite being a dangerous disease found across West Africa, with occasional cases imported elsewhere, few medical countermeasures for Lassa fever are available, with no vaccine or validated treatment, and limited regulatory-approved diagnostics. Scientific research on the Lassa virus (LASV) is needed to accelerate the development of such tools but is dependent on access to high-quality biological samples. To meet this need, we have established a well-curated biobank to prospectively collect, process, and store high-quality, well-characterized clinical specimens from patients with Lassa fever at the Federal Medical Center Owo (FMCO) in Nigeria. Patients presenting to FMCO with symptoms of Lassa fever were tested for LASV by polymerase chain reaction and screened for eligibility. Samples of whole blood, plasma, and serum were collected at baseline, and at 4- and 8-weeks post-baseline from LASV-positive (LASV+) participants. Of 197 participants enrolled, 99 were LASV+ and 98 LASV-negative. In total, 3,599 sample aliquots were stored, comprised of 264 whole blood, 1,572 serum, and 1,763 plasma samples. The Lassa fever biobank now provides support for scientific research, including the evaluation of diagnostics and biomarkers, as well as the development and validation of other medical countermeasures for the detection, prevention, and control of Lassa fever. Processes for accessing samples are described.

## INTRODUCTION

Lassa virus (LASV) is a zoonotic virus that causes a potentially severe acute hemorrhagic fever known as Lassa fever.^1^ Lassa fever is endemic in several West African countries, including Nigeria, Liberia, Guinea, and Sierra Leone, where it occurs both sporadically and as outbreaks.^2^^,^^3^ Several large-scale Lassa fever epidemics have occurred in Nigeria in recent years.^3^^,^^4^ In the first 15 weeks of 2023, 877 confirmed cases and 152 deaths were recorded, with a case fatality rate of 17%.^4^ The population at risk of Lassa fever is expected to increase over the next decades because of climate and land use changes.^5^

There are no validated therapeutics or vaccines for Lassa fever, although several products are under development.^1^^,^^6^^,^^7^ In addition, there are very few regulatory-approved LASV diagnostics appropriate for use in countries affected by Lassa fever, limiting disease surveillance.^8^^,^^9^ As such, LASV is a priority pathogen under the WHO Research and Development Blueprint—a platform for the acceleration of research and development for diseases that pose a public health risk because of their epidemic potential and for which available countermeasures are insufficient.^10^

Considering the lack of medical countermeasures and fundamental knowledge gaps regarding the epidemiology, immunology, and pathogenesis of Lassa fever,^11^ considerable research and development efforts are needed, including building capacity for disease surveillance, prevention, and control. The generation of this evidence is dependent on the availability of high-quality biological samples from individuals with or who recovered from Lassa fever. However, limited in-country collection, coordination, and service capacity, as well as a disconnect between demand and supply, pose major challenges to access to such specimens. Furthermore, LASV is a biosafety level 4 pathogen, presenting additional challenges to sample collection because of the requirement for maximum biological containment measures.

Certified biobanks of relevant, validated biological samples that have been correctly processed and stored are essential to accelerate the development of drugs, vaccines, and diagnostics.^12^^,^^13^ To meet this need, FIND and partners are building a network of integrated biobanks across Africa, Southeast Asia, South America, and Europe to improve access to high-quality samples for biomedical research.^14^ Samples are made available to qualified laboratories in academic and commercial organizations to facilitate research on Lassa fever, including the development and evaluation of new medical countermeasures.

The objective of the current project was to establish a well-curated biobank to prospectively collect, process, and store high-quality and well-characterized acute, convalescent, and negative specimens from patients suspected of having Lassa fever, as well as Lassa fever survivors, as part of the FIND Integrated Biobank (FIB) initiative.^14^

## MATERIALS AND METHODS

### Location.

A Lassa fever specimen biobank was established at the Federal Medical Center Owo (FMCO), in Ondo State, Nigeria. Ondo State is located in Southwest Nigeria and currently reports one of the highest proportions of confirmed Lassa fever cases in the country.^15^^–^^17^ This facility is a tertiary health facility and is one of the sites designated by the Nigeria Centre for Disease Control for Lassa fever testing, treatment, and research.

### Participants, study design, and ethics approval.

**Figure 1.**
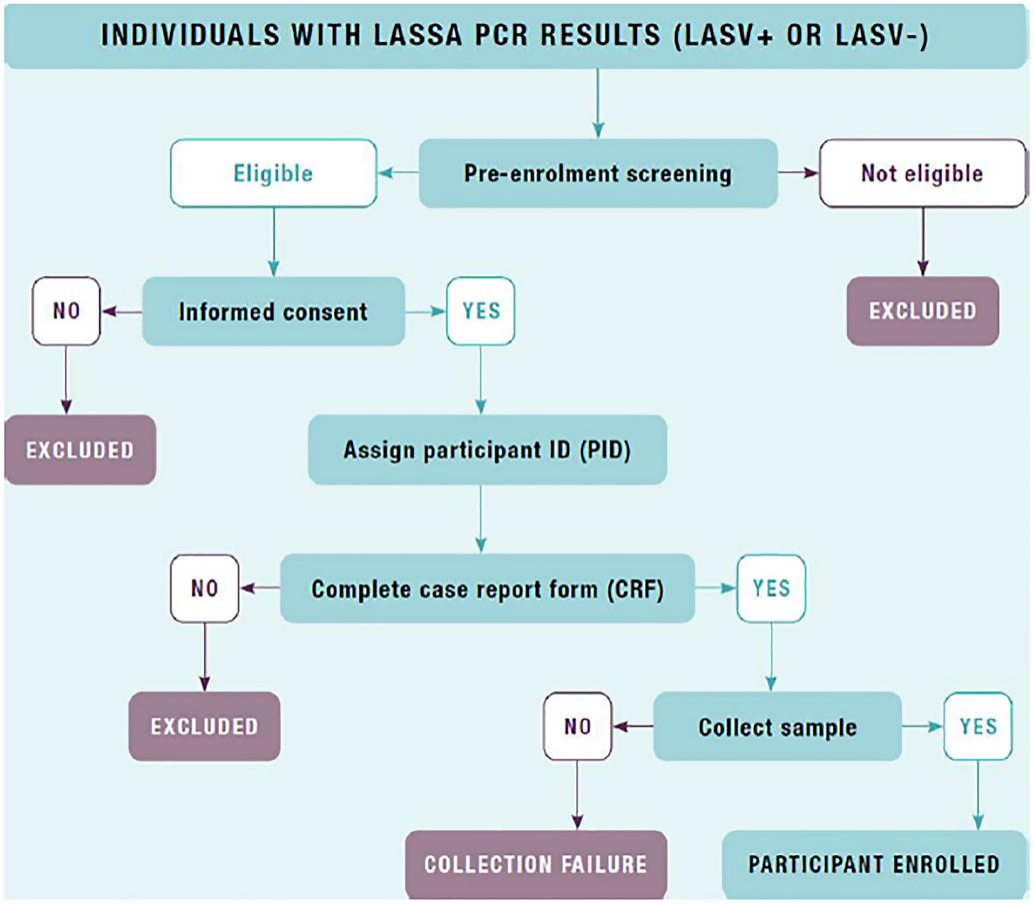
Screening process.

The diagnosis of Lassa fever in clinical suspects presenting to the FMCO outpatient and inpatient facilities is performed systematically using a combination of historical, clinical, and laboratory assessments following the National Guidelines for Lassa Fever Case Management.^18^ Laboratory confirmation on these clinical suspects was performed using Altona RealStar LASV qRT-PCR 2.0 (Altona Diagnostics, Hamburg, Germany), an in vitro real-time polymerase chain reaction (PCR) diagnostic test currently used as the gold standard for the qualitative detection of LASV-specific RNA,^19^ prior to screening for eligibility (Figure 1).

Participants were eligible for enrolment if they were ≥18 years of age or <18 years of age and qualified to provide either informed consent or informed assent accompanied by a parent or legal guardian, respectively. Individuals who were not competent to provide informed consent, as well as individuals with anatomical or health conditions contraindicating blood or bodily fluid collection, were excluded, as were participants for whom effective follow-up was deemed to be unachievable (e.g., those who lived elsewhere or were planning to move).

After the provision of informed consent and prior to sample collection, each participant underwent a clinical examination. Information collected included demographics, medical history, clinical measurements, treatment rendered, and clinical symptoms (e.g., specific symptom type and date of symptom onset). It is worth noting that these data were also collected from LASV+ participants at 4- and 8-week time points to better understand the disease progression in these individuals. All information was recorded by a trained nurse. Participant data were deidentified using unique participant identification numbers. Participants who were eligible and consented to participate but were not able to provide sufficient sample volume or enough samples per the protocol were considered collection failures and were not resampled. All samples were assigned a unique identification code before release to the biobank for storage.

Participant enrolment, as well as sample/data collection and management, were conducted in accordance with the established ethical principles derived from international guidelines, including the Declaration of Helsinki and applicable laws and regulations, including ISO 20387:2019, and good practices and guidelines, such as the International Council of Harmonization guidelines and WHO Good Clinical Laboratory Practice. Ethics approval (Ref: FMC/OW/380/VOL.CXIV/102, 13 April 2021) was obtained before the start of the study from the Health Research Ethics Committee of the Federal Medical Centre Owo, Nigeria.

### Specimen collection and processing.

**Figure 2.**
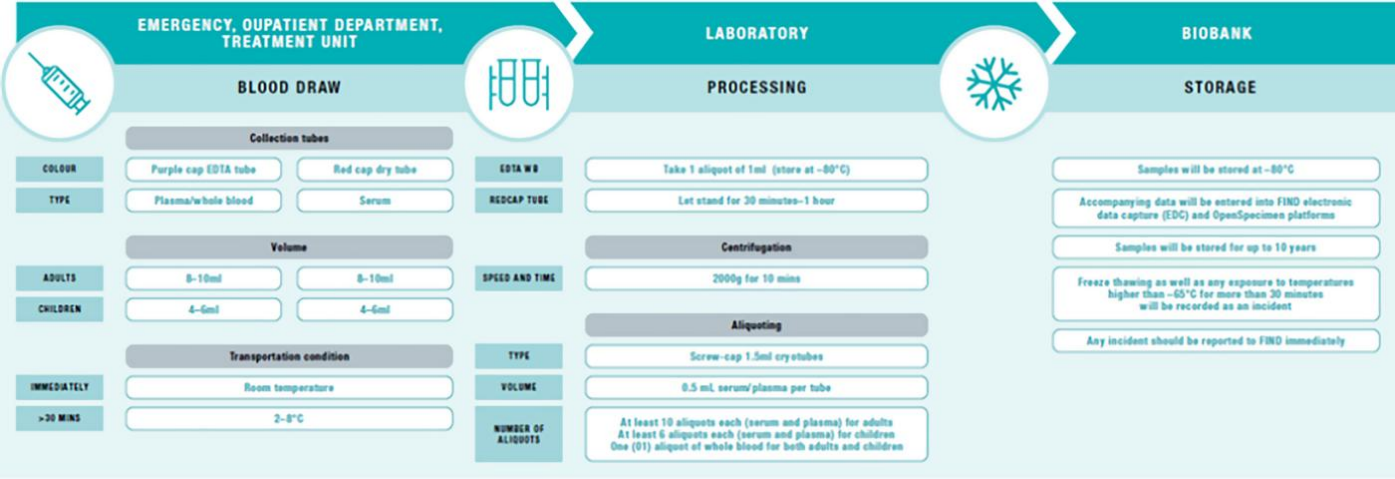
Specimen processing. EDTA = ethylenediaminetetraacetic acid.

Sample collection, processing, and storage details are shown in Figure 2. About 8–10 mL of whole blood were collected into red top blood tubes (i.e., containing only a clot activator) from adult participants and serum was decanted. Another 8–10 mL were collected into ethylenediaminetetraacetic acid (EDTA) tubes, from which 1 mL of whole blood was obtained and stored, and the remaining whole blood was processed into plasma via centrifugation. All blood processing was done using validated technical and safety standard operating procedures. From children, 4–6 mL of whole blood were collected in red top and EDTA tubes and processed into whole blood, serum, and plasma as described above for the adults. Baseline samples were collected at enrolment from each participant, as well as follow-up samples from LASV+ participants at 4 and 8 weeks to validate potential serological diagnostic tests relating to seroconversion. Aliquots for storage consisted of at least 10 0.5 mL aliquots for each serum and plasma sample for adults, six such aliquots for children, with a single 1 mL aliquot of whole blood for both adults and children. All samples were heat-inactivated at 56°C for 30 minutes before further processing, and all laboratory procedures were conducted in a Class III safety cabinet (Biobase, Budapest, Hungary), ensuring adherence to strict biosafety protocols to protect personnel and maintain sample integrity. Samples were stored consistently at –80°C.

### Data management.

Clinical data and sample information from baseline and follow-up visits were captured using OpenClinica (OpenClinica, LLC, Needham, MA) and OpenSpecimen (Krishagni Solutions Pvt Ltd., Pune, India) software, respectively. Data management was done in accordance with the International Council of Harmonization Good Clinical Practice principles, safeguarding the confidentiality of participants’ information.

## STATISTICAL ANALYSES

The target sample size was 200 (100 LASV+ and 100 LASV-negative [LASV–]) participants, to be recruited within 1 year. This sample size was determined according to the number of Lassa fever cases typically present at FMCO annually. Statistical analyses were performed on R Studio version 2023.06.1.

## RESULTS

Creation of the biobank facility began in March 2020 and enrolment of participants started in October 2021. Collection through September 2022 consisted of 197 participants (99 LASV+ and 98 LASV–) (Table 1). Ninety-nine (49.5%) participants were female between ages 20 and 40 years. The mean cycle threshold value on PCR was 30.2 for LASV+ participants, with no difference between females (30.9, SD = 4.4) and males (29.5, SD = 6.3) (*t(92) = 1.28, P* = *0.204*).

**Table 1:** Demographics of enrolled participants

Category	LASV PCR Positive, *n* (%)	LASV PCR Negative, *n* (%)	Total, *N* (%)
Total	99	98	197
Gender			
Females	46 (47)	53 (54)	99 (50)
Males	53 (53)	45 (46)	98 (50)
Age group			
<20	8 (8)	10 (10)	18 (9)
20–29	21 (21)	22 (22)	44 (22)
30–39	24 (24)	21 (21)	46 (23)
40–49	22 (22)	17 (17)	38 (19)
50–59	12 (12)	12 (12)	24 (12)
60–69	6 (6)	5 (5)	12 (6)
70–79	3 (3)	5 (5)	8 (4)
>80	3 (3)	6 (6)	7 (3.5)
Mean Ct value (SD)	30.2 (5.5)	–	–

Ct = cycle threshold; LASV PCR = Lassa virus polymerase chain reaction.

**Figure 3.**
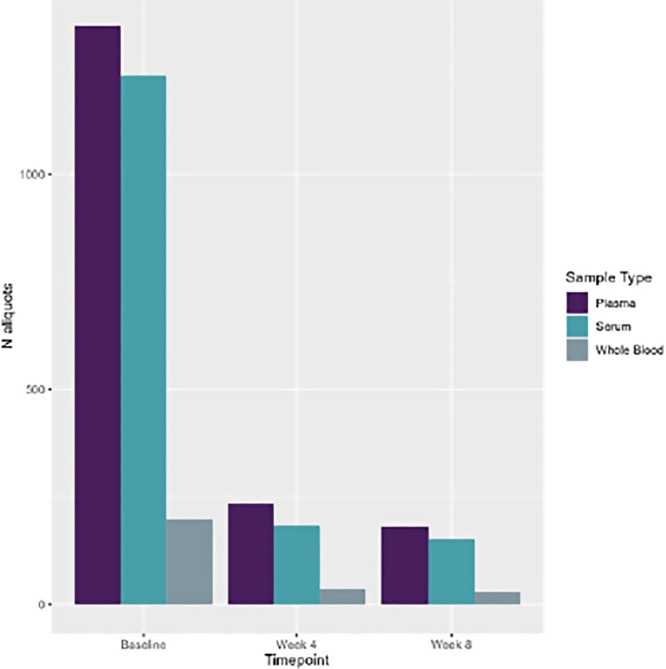
Sample distribution by type and events.

In total, 3,599 sample aliquots were collected, which comprised 264 whole blood, 1,572 serum, and 1,763 plasma samples (Figure 3). Of the 99 LASV+ participants, 36 (36%) had follow-up collections at 4 weeks, and 30 (30%) completed collections at both 4 weeks and 8 weeks follow-up. The leading cause of loss to follow-up was the participants’ unavailability at the time of the follow-up collection.

Since its establishment, the Lassa fever specimen biobank has received four sample requests, underscoring its relevance in advancing research and development. The requests have come from a diverse range of organizations, including two academic institutions conducting research, a public health institution, and a commercial entity focused on diagnostic and therapeutic development.

## DISCUSSION

The Lassa fever biobank was established in Nigeria, where the disease is endemic and frequent outbreaks continue to occur.^3^^,^^4^^,^^15^^,^^17^ All the required standard operating procedures, quality assurance, and governance processes for this well-curated biobank were established and implemented in compliance with the International Society for Biological and Environmental Repositories best practices.^20^ Within the framework of the FIB initiative, the collection and distribution of samples are mobilized by the local partner (FMCO). Available samples are listed on the DxConnect Virtual Biobank website, and FIND oversees all requests and ethical, governance, legal, and regulatory procedures. By offering funding, resources, and training to standardize services, FIND aims to empower the partner to operate independently while remaining interconnected to other partners within the network. Through this initiative, individual members’ capacity is strengthened through capacity-building and support while improving access to biological samples to address local and global research demands.^20^

Establishing biobanks in low- and middle-income countries and disease-relevant settings is essential for research and development to enable the necessary medical countermeasures and policies for their control. This may include the development of enhanced diagnostics to improve surveillance, therapeutics, and vaccines. The intended storage duration of samples in the biobank is 10 years. During this period, the samples will be made available to qualified laboratories in academic, nonprofit, independent, and private sector organizations for future research. To date, samples from the biobank have contributed to the evaluation of commercial LASV serological assays and a LASV antigen rapid test. The establishment and maintenance of biobanks in disease-endemic areas, with sample acquisition and scientific experimentation in those same areas, also obviates the need to overcome often complicated biosafety and biosecurity barriers increasingly inherent in sample shipment, which is especially relevant to high-consequence pathogens such as LASV. This approach also provides opportunities for local laboratory capacity building and for local communities to participate in the improvement of their public health conditions and contribute to general global health issues.

Biobanking of biosafety level 4 pathogens presents additional biosafety and resource considerations. A well-equipped and well-functioning infrastructure (including adequate laboratory facilities, equipment, power supply/backup, trained personnel, and standardized procedures) is essential for the proper collection, processing, and storage of biosafety level 4 samples. The resource requirements of such endeavors should be considered when securing funding, and sustainability considerations should be made for funding to maintain the biobank over the long term.

A major experience gained in establishing the Lassa fever biobank has been the importance of ethical and legal aspects and the unique considerations applicable to different communities and environments. These are paramount to ensure that collection and use of samples are conducted in a manner that ensures that the rights and well-being of participants are respected and that local regulations are considered for the successful undertaking of human subjects research. This includes appropriate informed consent processes, as well as systems structured to protect privacy and confidentiality. In addition, because the quality of the biological samples is crucial for successful research and development, biobanks should adhere to standardized guidelines and protocols for the collection, processing, and storage of the samples to ensure that sample integrity is maintained. Lastly, given that biobanking endeavors involve the collection and management of large amounts of data, robust data management systems should also be in place to ensure the accuracy, security, and privacy of the data.

The Lassa fever biobank provides access to high-quality, well-characterized specimens from LASV+ and LASV– individuals, covering the full course of disease progression. This will support the development, evaluation, and validation of diagnostic tests for Lassa fever while enabling broader research on biomarkers, disease pathogenesis, and immunological responses. Future project-specific collections may be conducted to address emerging scientific questions, such as host immune responses, viral evolution, or treatment efficacy. To ensure specimens are fit for purpose, rigorous quality assurance measures, standardized protocols, and an application-based access process have been implemented, ensuring their optimal use in research that advances Lassa fever diagnostics, therapeutics, and public health interventions. By facilitating access to high-quality, well-characterized clinical specimens, the biobank has contributed to various scientific endeavors, including the evaluation of serological and antigen-based diagnostic assays.

Lastly, interested parties can review FIND’s Dx Connect Virtual Biobank at https://vbd.finddx.org/ and request samples from the LASV biobank by contacting specimenbank@finddx.org. A review committee will evaluate sample requests and approve them based on supporting evidence, relevance to global health, technological adaptability, and affordability.
